# Dietary Interventions in Patients With Non-alcoholic Fatty Liver Disease: A Systematic Review and Meta-Analysis

**DOI:** 10.3389/fnut.2021.716783

**Published:** 2021-07-22

**Authors:** Veera Houttu, Susanne Csader, Max Nieuwdorp, Adriaan G. Holleboom, Ursula Schwab

**Affiliations:** ^1^Department of Vascular Medicine, Amsterdam University Medical Center, Location Amsterdam Medical Center at the University of Amsterdam, Amsterdam, Netherlands; ^2^Department of Experimental Vascular Medicine, Amsterdam University Medical Center, Location Amsterdam Medical Center at the University of Amsterdam, Amsterdam, Netherlands; ^3^School of Medicine, Institute of Public Health and Clinical Nutrition, The University of Eastern Finland, Kuopio, Finland; ^4^Department of Medicine, Endocrinology and Clinical Nutrition, Kuopio University Hospital, Kuopio, Finland

**Keywords:** non-alcoholic fatty liver disease, diet intervention, liver fat, liver transaminases, lipid metabolism, glucose metabolism, systematic review, meta-analysis

## Abstract

**Background:** With no approved pharmacotherapy to date, the present therapeutic cornerstone for non-alcoholic fatty liver diseases (NAFLD) is a lifestyle intervention. Guidelines endorse weight loss through dietary modifications, physical exercise, or both. However, no consensus exists on the optimal dietary treatment.

**Objectives:** The aim of our systematic review and meta-analysis was to summarize and assess the evidence for applied types of dietary interventions on the liver and metabolic outcomes in patients with NAFLD, aside from any effects of exercise intervention.

**Methods:** This systematic review was conducted according to the Preferred Reporting Items of Systematic Reviews and Meta-analysis (PRISMA) statement guidelines. The search was conducted in PubMed, Scopus, and Cochrane databases in February 2020. Included were only dietary interventions without exercise. This study was registered at PROSPERO: CRD42020203573.

**Results:** Eight randomized controlled trials, seven with endpoint reduction of hepatic steatosis, one with an assessment of endpoint fibrosis, were included in this systematic review, five of which were included in the meta-analysis. Mediterranean dietary interventions without energy restriction (*n* = 3) showed significant reduction of intrahepatic lipid content (IHL) (SDM: −0.57, 95% CI: −1.04, −0.10), but there was no significant change in alanine transaminase (ALT) (SDM: 0.59, 95% CI: −0.5, −1.68). Hypocaloric dietary interventions with foods high in unsaturated fatty acids (*n* = 2) led to a significant decrease in ALT (SDM: −1.09, 95% CI: −1.49, −0.69) and aspartate aminotransferase (AST) (SDM: −0.75, 95% CI: −1.27, 0.23); yet effects on steatosis could not be aggregated due to different assessment techniques. Mediterranean diet did not lead to significant changes in concentrations of gamma-glutamyl transpeptidase (γGT), total cholesterol (TC), low-density lipoprotein cholesterol (LDL-C), high-density lipoprotein cholesterol (HDL-C), triglyceride (TG), fasting glucose or insulin, or homeostatic assessment for insulin resistance.

**Conclusions:** In patients with NAFLD, Mediterranean and hypocaloric dietary interventions favoring unsaturated fatty acids result in improvements in IHL and transaminases. Since many dietary intervention studies are combined with exercise interventions and there is a paucity of ample-sized studies examining dietary interventions on the more advanced and clinically relevant stages of NAFLD, that is active and fibrotic NASH, with multiparametric imaging and liver histology as outcome measures, the optimal dietary invention in NAFLD remains to be defined.

## Introduction

Non-alcoholic fatty liver disease (NAFLD) has become the most common cause of chronic liver disease worldwide (1–3). Currently, 25% of the global and 90% of the obese population have some degrees of NAFLD. The global prevalence of its active stage, non-alcoholic steatohepatitis (NASH), is ~3–5% (2). NAFLD comprises a spectrum of liver disease, clinically defined as more than 5% hepatic fat accumulation excluding significant alcohol consumption and other hepatic diseases (4, 5). It ranges from benign steatosis to NASH, characterized by hepatocellular inflammation and ballooning, which can subsequently result in hepatic fibrosis (6, 7). Fibrotic NASH can progress to cirrhosis and it can lead to hepatocellular carcinoma (HCC), even at the precirrhotic stage. Indeed, several studies have found a strong relation of NAFLD fibrosis stage with liver-related morbidity and mortality and also with all-cause mortality (8, 9). Several driving factors for a progressive NAFLD course have been recognized. In addition to obesity, insulin resistance and the related metabolic syndrome (MetS), and Type 2 diabetes mellitus (T2DM) are strongly related to NAFLD. Approximately 60% of patients with T2DM have NAFLD (10–12). Consequently, patients with T2DM have a 2.5-fold higher risk of mortality due to chronic liver disease than non-diabetics (13), and this risk is elevated in MetS as well (14). NAFLD often gives mixed hyperlipidemia with elevated concentrations of low-density lipoprotein cholesterol (LDL-C), decreased levels of high-density lipoprotein cholesterol (HDL-C), and triglycerides (TGs); over 50% of patients with dyslipidemia have NAFLD (10, 15). In addition to obesity and T2DM, high-calorie intake, a Western-type diet, a sedentary lifestyle, sleep apneas, and low-grade inflammation arising from gut microbial dysbiosis or inflammatory conditions, such as psoriasis contribute to drive progression along the NAFLD spectrum (16–20).

Cornerstone interventions in halting or reversing NAFLD progression are lifestyle changes toward healthy diet and increased physical activity (21–24). Several international guidelines for the clinical management of NAFLD endorse weight loss either through the combination of dietary modifications and physical activity or either through one of these two alone. A reduction of hepatic steatosis can already be achieved with a weight loss of 5–7%, and to improve fibrosis and inflammation, more than 10% of weight loss is suggested (25, 26). Several dietary factors, such as increased intake of refined carbohydrates, in particular, fructose and sucrose and foods high in saturated fat, such as processed meat or high fat dairy have been associated with the development of NAFLD (27–29). On the contrary, dietary fiber and unsaturated fatty acids have been shown to reduce *de novo* lipogenesis, improve insulin sensitivity, increase satiety, and modulate gut microbiota attenuating the development or the onset of NAFLD (29, 30). However, there is still no consensus of the macronutrient composition for the dietary modifications for patients with NAFLD. Whereas, the European Association for the Study of the Liver (EASL), European Society for Clinical Nutrition and Metabolism (ESPEN), and American Gastroenterology Association (AGA) propose the Mediterranean style diet, the American Association for the Study of Liver Disease (AASLD) does not directly recommend a particular diet, although the Mediterranean diet is mentioned (21–24). Moreover, recently published systematic reviews (SRs) on dietary modifications include studies with dietary interventions combined with physical activity (31, 32). Therefore, the evidence on the effects of specific dietary approaches alone on NAFLD seems poorly aggregated. Of note, none of the SRs studied the effect of dietary modifications on the liver inflammation or fibrosis, the progressive stages of NAFLD related to morbidity and mortality.

In this systematic review, we aimed to review randomized controlled trials (RCTs) with dietary interventions without any exercise or physical activity intervention in patients with NAFLD in order to establish the effect of different dietary modifications on intrahepatic lipid content (IHL), liver fibrosis, and liver function in patients with NAFLD. Additionally, the effects of dietary interventions on body weight, glucose metabolism, and plasma lipid profile were reviewed. This work is of great value due to the inclusion of dietary intervention studies *per se*, that is, without exercise or physical activity interventions, in well-characterized study populations.

## Methods

The present SR was performed based on the Preferred Reporting Items of Systematic Reviews and Meta-analysis (PRISMA) statement guidelines (33, 34). Patient, intervention, comparison, and outcome (PICO) statement was applied in the process. The protocol of this systematic review was registered in PROSPERO database (CRD42020203573).

### Search Strategy and Data Sources

For gathering all RCTs investigating the effect of dietary interventions without exercise intervention on patients with NAFLD, a systematic literature search was performed by an information specialist together with the authors. The search in Pubmed, Scopus, and Cochrane database for clinical trials were conducted until February 2020 by using the keywords, such as “non-alcoholic fatty liver,” “NAFLD,” “NASH,” “steatohepatitis,” “diet,” “nutrition,” “liver enzymes,” “liver fat,” “blood lipids,” and “glucose metabolism.”

### Eligibility Criteria: Inclusion and Exclusion

Studies were included when they met the following conditions: (1) RCTs written in English, (2) isocaloric or hypocaloric dietary interventions without exercise intervention and changes in alcohol consumption, (3) a minimum intervention period of 4 weeks, (4) studies conducted on adults (age ≥18 years), (5) diagnosis of NAFLD made either based on histology, ultrasonography (US) including vibration-controlled transient elastography (VCTE) (FibroScan®, Echosens, Paris, France.), MRI, or computed tomography (CT), (6) presence of a control group with unchanged or control dietary intervention. Excluded studies were (1) non-human, studies with adolescents, pilot, prospective cohort, and cross-sectional and uncontrolled studies; (2) abstracts, reviews, and case reports; (3) hypercaloric dietary interventions, studies with specific food items, or dietary regimens.

### Screening Process

Two reviewers (SC and VH) screened the articles independently based on title and abstract using the Rayyan QCRI program (35). Duplicates were removed. All studies which did not fit into our inclusion and/or exclusion criteria were excluded. After screening for eligibility, full-text articles were resorted independently by the same authors. If agreement could not be reached, US was consulted.

### Quality Assessment

Quality assessment was performed for all eligible studies independently by SC and VH using the quality assessment tool for clinical trials (36). The tool has a three-category grading system of the Agency for Healthcare Research and Quality (AHRQ) and it is specifically designed to dietary intervention studies. It includes detailed sub-questions under the following main scoring items: (1) general question and study design, (2) participants and compliance, (3) dietary intervention and assessment, (4) outcome, results, and analysis and (5) summary of the study quality. Evaluated studies obtained A-, B-, or C-levels based on the answers, “yes,” “no,” “cannot tell,” or “not applicable” to the sub-questions. Studies rated Level A had the least bias and results were considered valid whereas Level B studies were susceptible to some concern, but not sufficient to invalidate the results. C-level rating indicated significant bias that may invalidate the results in studies.

### Data Extraction

After the screening process and quality assessment, data were extracted from eligible studies independently by the two reviewers (SC and VH). Extracted data included the outcome variables for the following factors: (1) intrahepatic fat content (IHL) and (2) fibrosis and liver function including (3) alanine aminotransferase (ALT) and (4) aspartate aminotransferase (AST), (5) gamma-glutamyl transferase (γGT), glucose metabolism markers including (10) fasting glucose, (11) fasting insulin, (12) homeostatic model assessment for insulin resistance (HOMA-IR), and plasma lipid profile parameters including (6) total cholesterol (TC), (7) LDL-C, (8) HDL-C, and (9) TG.

### Statistical Analysis

For meta-analyses, R programming software (Version 4.0.3) was used. Changes in mean and standard deviation (SD) before and after the intervention were calculated for each parameter according to the Cochrane Handbook for Systematic Reviews of Interventions (37). Publication-bias detection analyses could not be performed due to lack of power. Meta-analysis was performed for the following outcomes: IHL, ALT, AST, γGT, fasting glucose, fasting insulin, HOMA-IR, TC, LDL-C, HDL-C, and TG. All continuous parameters were displayed as standardized mean difference (SMD) to measure the effect size and were presented with 95% confidence interval (CI). Fixed or random effect model was used to pool and calculate the SMD from baseline to follow-up between intervention and control group. *I*^2^ was used to assess the heterogeneity across the studies. Low heterogeneity was considered if *I*^2^ was <25% and the fixed effect model was applied for the meta-analysis. In case of moderate (25–50%) and high heterogeneity (>50%), the random effect model was used.

## Results

### Database Search and Study Selection

**Figure 1:**
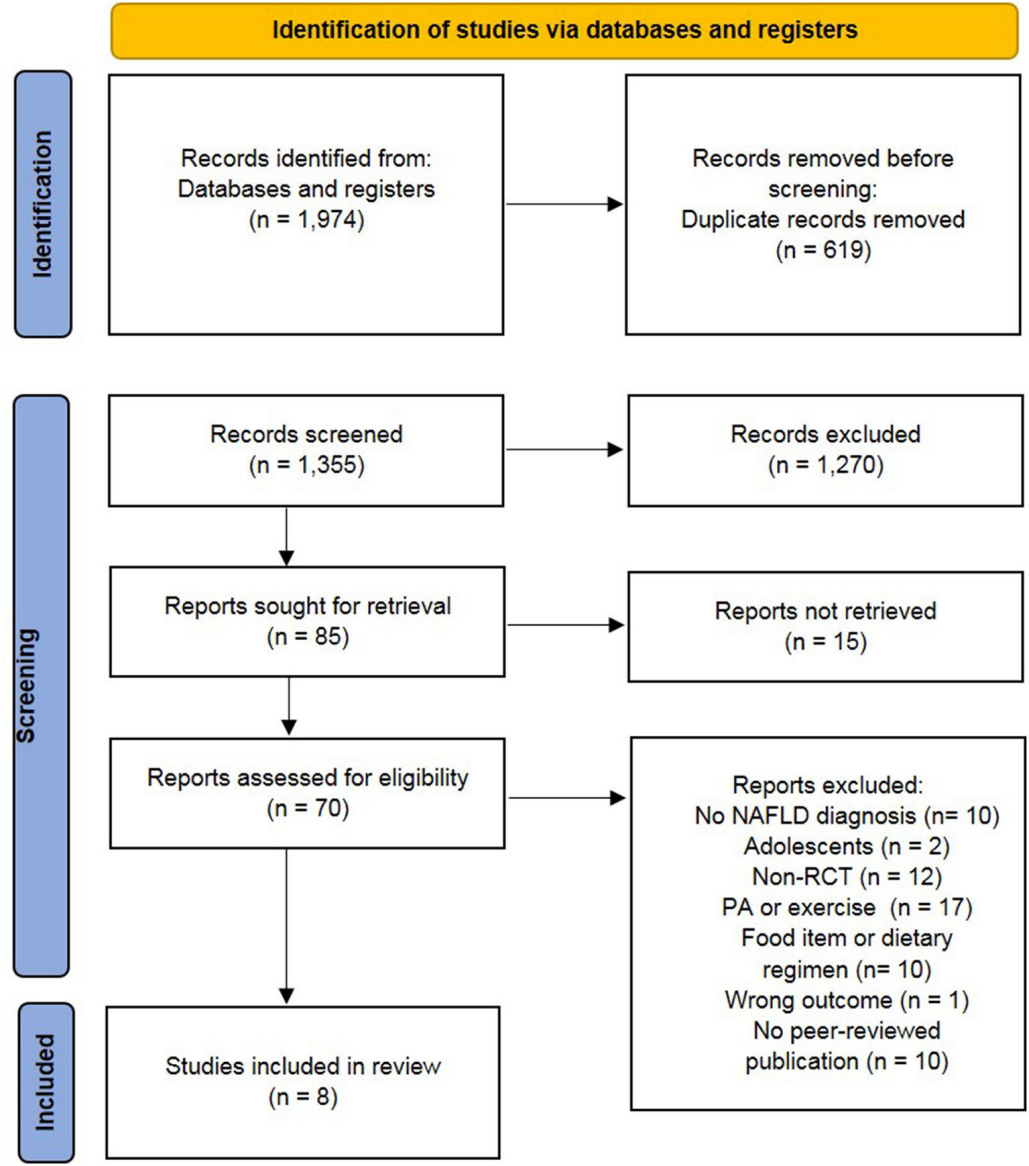
Preferred reporting items of systematic reviews and meta-analysis (PRISMA) flow chart (34). NAFLD, non-alcoholic fatty liver diseases; non-RCT, non-randomized controlled trial; PA, physical activity.

Primary search yielded 1,974 records. After the removal of the duplicates, 1,355 were screened based on the abstract and title, and 85 studies were retained for full-text assessment. According to the inclusion and exclusion criteria, eight studies fulfilled the criteria for further analyses including qualitative synthesis. Five of these studies were included in the meta-analysis. All stages of the study selection process are presented in the flow chart (Figure 1).

### Quality Assessment

The quality assessment results are presented in Table 1. Three studies have low risk of bias and the results are considered valid (42, 44, 45). Some concerns appeared in four studies, mostly because of missing reported randomization methods (39–41, 43). In one of the studies, a significant bias was found not only due to concerns in the study design but also due to the dietary assessment (38).

**Table 1:** Characteristics and quality assessment results of the included studies.

**Author, year of publication and country**	**Sample size (F/M)**	**Mean age (years ± SD)**	**Disease stage**	**Diagnosis techniques**	**Outcome measurement technique**	**Duration (week)**	**Quality assessment^**a**^**
Arefhosseini et al. (38), Iran	I: 22 (10/12) C: 22 (12/10)	I: 38.0 ± 8.1C: 40.6 ± 8.3	Steatosis grade I–III	US	US	6	C
Jang et al. (39), South Korea	I: 52 C: 54	I: 43.6 ± 11.8C: 42.4 ± 3.0	NA	US	CT	8	B
Razavi et al. (40), Iran	I: 30 (15/15) C: 30 (15/15)	I: 42.8 ± 10.6C: 39.7 ± 7.3	Steatosis Grade I–III	US	US	8	B
Shidfar et al. (41), Iran	I:25 (8/13) C:28 (9/13)	I: 46.1 ± 8.4C: 45.7 ± 0.8	Steatosis mild to moderate	Increased levels of AST^*^/ALT^*^	US	12	B
Dorosti et al. (42), Iran	I: 47 (21/26) C: 47 (29/18)	I: 43.1 ± 8.9C: 42.4 ± 8.6	Steatosis Grade I–III	US	US	12	A
Misciagna et al. (43), Italy	I: 39 (13/26) C: 43 (10/33)	I: 49.3 ± 10.4C: 54.1 ± 1.4	NAFLD moderate to severe	US	US	24	B
Propezi et al. (44), Australia	I: 24 (11/15) C: 24 (14/11)	I: 51.0 ± 13.4C: 53.0 ± 9.1	Steatosis Grade I–III	MRS-PDFF	MRS and Fibroscan	12	A
Ryan et al. (45), Australia	12 (6/6)	55.0 ± 14.0	11.2 ± 2.1% IHL	Liver histology and US	MRS	6	A

*C, control group; CT, computed tomography; F, female; I, intervention group; M, male; MRS, magnetic resonance spectroscopy; MRS-PDFF, magnetic resonance spectroscopy proton density fat fraction; NAFLD, non-alcoholic fatty liver disease; NA, not available; RCT, randomized controlled trial; SD, standard deviation; US, ultrasonography*;

* *reference ALT/AST (<30 IU/L male, <20 IU/L female)*.

a *Quality assessment tool for clinical trials of the Agency for Healthcare Research and Quality (AHRQ)*.

### Study Characteristics

In this systematic review, the sample size of the included studies varied from 12 participants (45) to 112 participants (42). In total, the studies performed interventions with 499 participants with NAFLD. The study duration varied from 6 weeks (38, 45) to 24 weeks (43). Three studies had an intervention duration of 12 weeks and two studies were 8-week interventions (39, 40). In seven studies, the population consisted of both females and males. One study (39) did not report the gender distribution. The mean age ranged from 38 years (38) to 55 years (45). Most of the studies used US or magnetic resonance spectroscopy (MRS) to diagnose and stage NAFLD, and hence classified steatosis only, ranging from steatosis Grade I to Grade III (38–44). Signs of NASH as elevated transaminases, ALT and/or AST, were assessed in six studies (38–41, 44, 45). One study reported hepatic fibrosis as liver stiffness measurement (LSM) by VCTE (FibroScan®) (44). One of the studies also had subjects with histologically confirmed NAFLD, where IHL was found to be 11.2 ± 2.1% (45). The characteristics of the included studies are presented in Table 1.

### Dietary Interventions

Dietary interventions (Table 2) and the duration of interventions (Table 1) varied between the studies. Four out of eight studies conducted a hypocaloric dietary intervention (38–41), whereas isocaloric diets were followed in four studies (42–45). However, due to high variations in the dietary interventions, only two studies with a hypocaloric dietary intervention (40, 41) and three studies with an isocaloric dietary intervention (42–45) could be selected for a meta-analysis based on the dietary intervention regimen.

**Table 2:** Macronutrient composition, content of food items, and energy content of the intervention and control diets of the included studies.

**References**	**Intervention diet (macronutrient composition, food items, and energy content kcal/d)**	**Control diet (macronutrient composition, food items, and energy content kcal/d)**
**Hypocaloric dietary interventions**		
Arefhosseini et al. (38)	Diet 1 Carbohydrate: Fat: Protein: 55: 25: 20 −500 kcal/d	Diet 2 Carbohydrate: Fat: Protein: 40: 40: 20 −500 kcal/d
Jang et al. (39)	Low-carbohydrate diet Carbohydrate: Fat: Protein: 50–60:20–25:20–25 25 kcal/kg of ideal body weight	Low-fat diet Carbohydrate: Fat: Protein: 60–70:15–20:15–20 25 kcal/kg of ideal body weight
Razavi et al. (40)	DASH diet high in fruits, vegetables, whole grains, and low-fat dairy products and low in saturated fats, cholesterol, refined grains, sweets, and sodium <2,400 mg/day. Carbohydrate: Fat: Protein: 52–55: 30: 16–18 −350–700 kcal based on the computed energy requirement per subject and BMI.	Control diet with fewer vegetables and fruits, less seeds and legumes, less low-fat diary, less whole-grains, and more simple sugars.Carbohydrate: Fat: Protein: 52–55: 30: 16–18−350–700 kcal based on the computed energy requirement per subject and BMI.
Shidfar et al. (41)	Carbohydrate: Fat: Protein: 50: 30: 20; where 30% of fat from olive oil and 10% from other sources (dairy, meats, and nuts). Target 5% weight reduction based on calculation on age, weight, and height.	Carbohydrate: Fat: Protein: 50: 30: 20; where 30% of the fat was culinary fat and 10% from other sources (dairy, meats, and nuts).Target 5% weight reduction based on calculation on age, weight, and height.
**Isocaloric dietary interventions**		
Dorosti et al. (42)	Whole-grain diet (List and description of whole-grain foods and advises to consume at least half of the daily grain servings as whole grains based on Dietary Guidelines for Americans 2012. Two to three servings of low-fat dairy products, five serving of fruits and vegetables, and two servings of lean meat, poultry, or fish daily).	Consumption of usual cereals. Two to three servings of low-fat dairy products, five serving of fruits and vegetables, and two servings of lean meat, poultry, or fish daily.
Misciagna et al. (43)	Low glycemic index Mediterranean diet. Carbohydrate: Fat: Protein: 50: 30 (<10% saturated fat, and MUFA and PUFA from olive oil, plant and marine sources): 20	Control diet (based on the Italian National Research Institute for Foods and Nutrition Guidelines)Carbohydrate: Fat: Protein: 40: 35–40 (<10 % saturated fat): 20
Propezi et al. (44)	Mediterranean diet (based on the data on the consumption of foods in the traditional Cretan diet with alterations to protein intake to be standardized with the low-fat diet). Carbohydrate: Fat: Protein: 40: 35–40 (MUFA and n-3-PUFA): 20	Low-fat diet (based on Australian National Heart Foundation Diet and the American Heart Association Diet).Carbohydrate: Fat: Protein: 50: 30: 20
Ryan et al. (45)	Mediterranean diet (based on the reported data on the traditional Cretan diet including meals and meal-based traditional recipes and food preparation techniques, and foods, such as olives, dried fruits, nuts, Greek yogurt, fish, and extra virgin olive oil). Carbohydrate: Fat: Protein: 40: 40 (MUFA and n-3-PUFA from plant and marine sources): 20	Low-fat/high-carbohydrate diet (based on the Australian National Heart Foundation Diet and the American Heart Association Diet).Carbohydrate: Fat: Protein: 50: 30 (n-6-PUFAs): 20

*BMI, Body Mass Index; DASH, Dietary approaches to stop hypertension diet; kcal/d, kilocalories per day; MUFA, monounsaturated fatty acid; n-3-PUFA, omega-3 polyunsaturated fatty acid; n-6-PUFA, omega-6 polyunsaturated fatty acid*.

Two studies with hypocaloric dietary intervention were selected for meta-analysis with the focus on the effect of dietary fat intake since both have used foods or dietary pattern high in unsaturated fatty acids compared to their controls. Razavi et al. (40) introduced dietary approaches to stop hypertension (DASH) diet, which is a dietary pattern with restricted intake of salt and salt-containing products, increased consumption of vegetables, fruits, and whole-grain products, as well as dietary fats containing unsaturated fatty acids (46). In the study by Shidfar et al. (41), the intervention diet consisted of a diet with a macronutrient composition toward increased intake of unsaturated fatty acids from olive oil, while the control group had the intake of fat from other unspecified culinary dietary fats. The other two hypocaloric dietary studies by Arefhosseini et al. (38) and Jang et al. (39) could not be integrated in the meta-analysis for comparisons of macronutrient proportions, since the proportions of macronutrients were not comparable between these two.

For the isocaloric studies, three studies were included in the meta-analysis. These studies conducted a Mediterranean dietary intervention with varying macronutrient compositions (43–45), and had as a control group, a low-fat low-carbohydrate diet based on dietary guidelines. In the study by Ryan et al. (45), subjects undertook both diets according to a cross-over design with a washout period of 6 weeks. The Mediterranean diet in these studies mainly consisted of vegetables, fruits, legumes, nuts, and unsaturated dietary fats from vegetable oils. The study by Dorosti et al. (42), which was not included in the meta-analysis, focused on the effect of whole-grain diet compared to the usual cereal and grain products on NAFLD. Characteristics of macronutrient composition, energy content, and food choices of the dietary interventions are described in Table 2.

### Meta-Analysis

For hypo-caloric studies, meta-analyses were conducted for the liver function including ALT and AST (40, 41). Analysis could not be performed for IHL, lipid, or glucose parameters in this group due to lack of studies. For the isocaloric group (the Mediterranean diet), meta-analysis could be performed in IHL, ALT, and γGT, as well as for the glucose parameters, such as fasting glucose, fasting insulin, and HOMA-IR, and for the lipid parameters, such as TC, HDL-C, LDL-C, and TG. The ALT was measured only in two studies (44, 45), and AST was not analyzed because it was measured in only one study (42). Two studies analyzed TC, LDL-C, and HOMA-IR (43, 44), and for γGT, HDL-C, TG, fasting glucose, and insulin, all the three included studies measured these parameters (43–45). Meta-analysis for fibrosis status could not be performed since only one study assessed fibrosis with LSM, a proxy for fibrosis (44). However, paired LSM did not show any significant change after the diet intervention between the intervention and the control group. In another study, the diagnosis of NAFLD by the liver biopsy together with US was an inclusion criterion, but the liver biopsy was not repeated after the intervention as an outcome measure of the intervention (45). Altogether, none of the studies assessed the liver histology or used multiparametric imaging in order to establish the effects of dietary interventions on the active or fibrotic NASH.

### Liver Parameters

All studies measured IHL, but different methods were used to assess the liver fat content Table 1. In the hypocaloric group, Arefhosseini et al. (38) reported a significant reduction of hepatic steatosis grade in both groups (intervention: *p* = 0.011; control: *p* = 0.014) and Jang et al. (39) showed significant reduction of the liver fat in the low carbohydrate group (intervention) compared to the low-fat control group (the liver/spleen HU ratio: 0.85 vs. 0.92, *p* = 0.015). Razavi et al. (40) showed significant reduction in the percentage of NAFLD grade within both groups (intervention: *p* < 0.001, control: *p* < 0.001). Shidfar et al. (41) reported a reduction of IHL between the baseline and after the olive oil dietary intervention (*p* = 0.008), but it did not differ from the reduction in the control group. All studies in the isocaloric group reported a decrease in IHL, compared to controls (42–45). Dorosti et al. (42) found a significant decrease in the hepatic steatosis grade in the whole-grain group (*p* < 0.001)and Propezi et al. (44) reported a significant reduction in IHL in both groups (intervention: *p* < 0.001, control: *p* < 0.001), but not between the groups after the intervention. The IHL reduction upon Mediterranean diet (−5.6 ± 7.4 %, *p* < 0.005) was significantly higher compared to the low-fat control group (−1.2 ± 2.6 %, *p* > 0.05) in the study by Ryan et al. (45). In line with this, between the groups, a significant relative reduction of IHL favoring Mediterranean diet was reported (intervention: 39 ± 4% vs. control: 7 ± 3%, *p* = 0.012).

**Figure 2:**
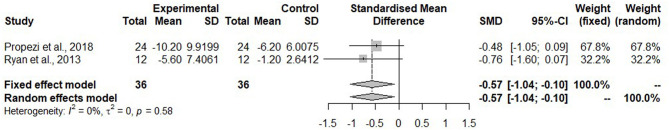
Forest plot of intrahepatic liver fat (IHL); SD, standard deviation; SMD, standard mean difference.

For the meta-analysis, only studies which reported IHL as mean percentage using MRI were considered. These were two studies (43, 44) in the isocaloric group with the Mediterranean diet. The IHL was significantly reduced after the Mediterranean diet compared to the control diet (SMD: −0.57, 95% CI: −1.04, −0.10) (Figure 2). Heterogeneity was not detected among the studies (*I*^2^ = 0%, τ^2^ = 0, *p* = 0.58).

The liver enzymes, ALT and AST, imperfect by clinically widely used proxies for steatohepatitis, were measured in most of the studies, whereas γGT was measured only in five studies (39, 42, 44, 45). Arefhosseini et al. (38) reported a significant reduction of these liver enzymes in both groups (intervention: −9.09 ± 12.8 IU/L, *p* = 0.020; control: −8.91 ± 10.0 IU/L, *p* = 0.009). Jang et al. (39) (intervention: −30.47 ± 26.0 IU/L, *p* < 0.001) and Razavi et al. (37) (intervention: −8.4 ± 12.7 IU/L, *p* = 0.02) found a significant reduction of ALT compared to the control group. In addition, Jang et al. (39) reported a significant reduction of AST (−12 ± 17.4 IU/L, *p* < 0.001) in the low carbohydrate intervention and Razavi et al. (40) found the reduction of AST in the DASH diet (−10.7 ± 23.1 IU/L, *p* = 0.01). Shidfar et al. (41) showed a significant reduction of ALT in both groups (intervention: −12.29 ± 7.8 IU/L, *p* < 0.001; control: −4.64 ± 6.5 IU/L, *p* = 0.004) as well as AST in the intervention group (−8.43 ± 3.4 IU/L, *p* < 0.001). In the study by Dorosti et al. (42), all liver enzymes significantly decreased in the group with whole-grain foods compared to the control group (ALT: −22.4 ± 7.8 vs. 0.4 ± 11.17 IU/L, *p* < 0.001; AST: −5.8 ± 9.1 vs. 1 ± 6.6 IU/L, *p* < 0.001, γGT: −4.5 ±7.2 vs. 1.7 ± 12.3 IU/L, *p* = 0.009). For the Mediterranean diets, Propezi et al. (44) detected significant changes of ALT (intervention: −8.0 ± 31.2 IU/L, *p* = 0.004; control: −12 ± 40.4 IU/L, *p* = 0.049) in both groups and γGT (intervention: −19 ± 72.1 IU/L, *p* < 0.001) only in the intervention group.

**Figure 3:**
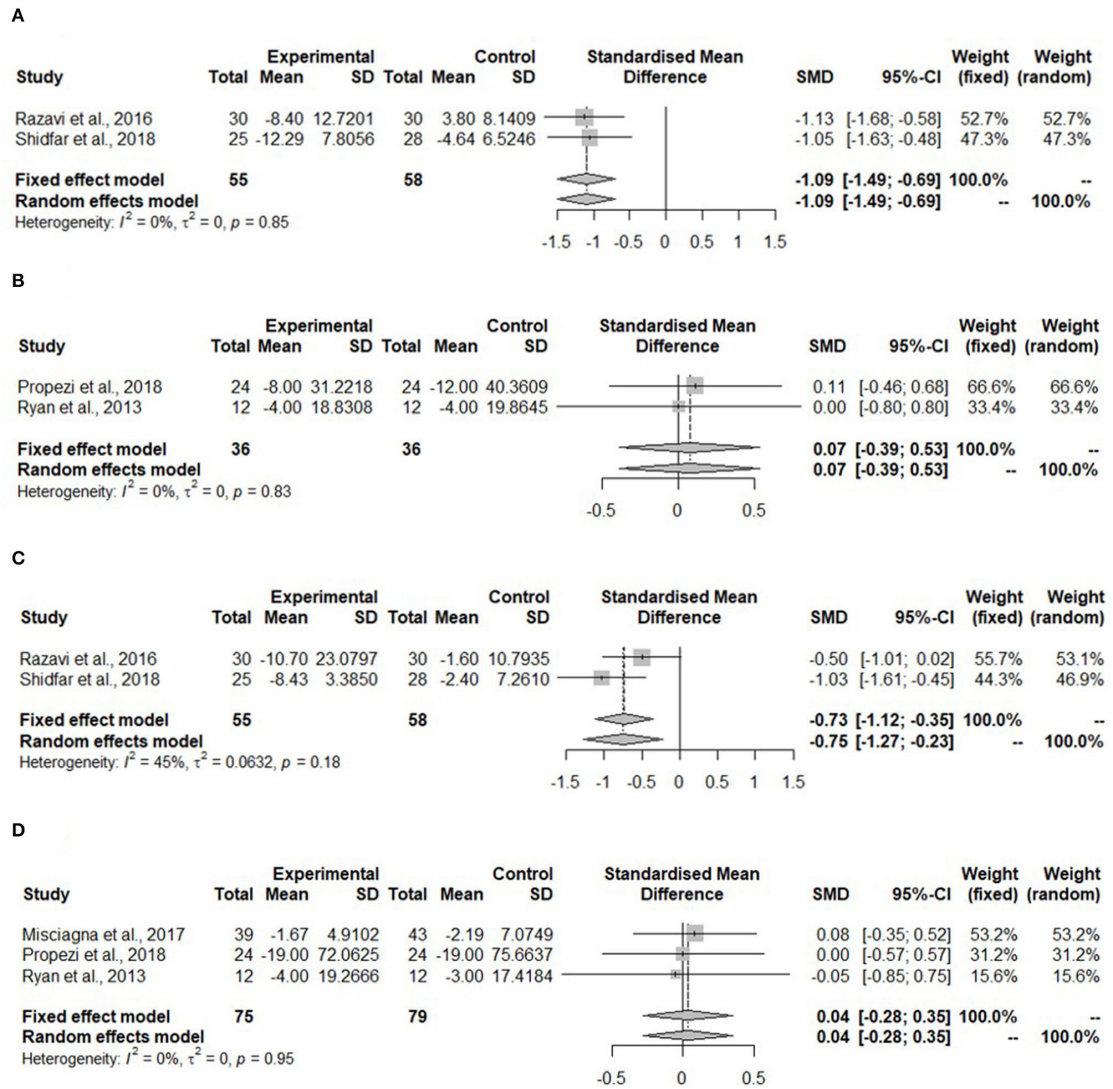
Forest plots of **(A)** alanine aminotransferase (ALT) in hypo-caloric group; **(B)** ALT in isocaloric group; **(C)** aspartate aminotransferase (AST) in hypocaloric group; **(D)** gamma-glutamyl transferase (γGT) in isocaloric group; SD, standard deviation; SMD, standardized mean difference.

Figure 3A shows the meta-analysis of ALT in the hypocaloric group. The ALT was significantly reduced upon dietary intervention compared to the control group (SMD: −1.09, 95% CI: −1.49, −0.69) and heterogeneity was not found (*I*^2^ = 0%, τ^2^ = 0, *p* = 0.85). For the Mediterranean diet, two studies reported ALT (Figure 3B). The ALT did not change (SMD: 0.07, 95% CI: −0.39, 0.53). The heterogeneity in the effect of the Mediterranean diet was not detected (*I*^2^ = 0%, τ^2^ = 0, *p* = 0.83).

Aspartate aminotransferase was measured in the study with whole-grain dietary intervention by Dorosti et al. (42), but it was not measured in the Mediterranean diet intervention studies. No meta-analysis could be performed. For the hypocaloric interventions, both studies measured AST (Figure 3C). This kind of diet reduced AST significantly compared to the control group (SMD: −0.75, 95% CI: −1.27, 0.23). The heterogeneity was moderate, but it was not significant (*I*^2^ = 45%, τ^2^ = 0.0632, *p* = 0.518).

The gamma-glutamyl transferase was not measured in any of the hypocaloric studies, but it was measured in all of the Mediterranean diet interventions. Figure 3D shows that the concentration of γGT was not changed after the intervention compared to the control group (SMD: 0.04, 95% CI: −0.28, 0.35) and no heterogeneity was detected (*I*^2^ = 0%, τ^2^ = 0, *p* = 0.95).

### Glucose Parameters

Only Jang et al. (39) and Razavi et al. (40) in the hypocaloric group reported fasting glucose and insulin concentrations. Razavi et al. (40) measured HOMA-IR as well. Only Razavi et al. (40) reported significant changes for insulin (−3.3 ± 3.2 μIU/mL, *p* < 0.001) and HOMA-IR(−0.8 ± 0.9 μIU/ml, *p* < 0.001) in the DASH diet. Ryan et al. (45) showed a reduction of glucose concentration for both interventions. In the isocaloric group, Dorosti et al. (42) showed a decrease in insulin (−2.1 ± 5.6 mU/L, p < 0.05) and in HOMA-IR (−0.5 ± 0.2, *p* < 0.05) in the whole-grain intervention diet. However, after adjusting for baseline values of the outcome, the mean change in food groups and metabolic equivalent of task (MET) value, no significant changes were observed. Misciagna et al. (43) showed a reduction in the glucose and insulin concentration in both intervention groups, but no statistical analysis was performed on these results. Ryan et al. (45) detected a significant decrease in insulin (−6.7 ± 5.3 mIU/L, *p* < 0.01) in the Mediterranean diet compared to the low-fat diet.

**Figure 4:**
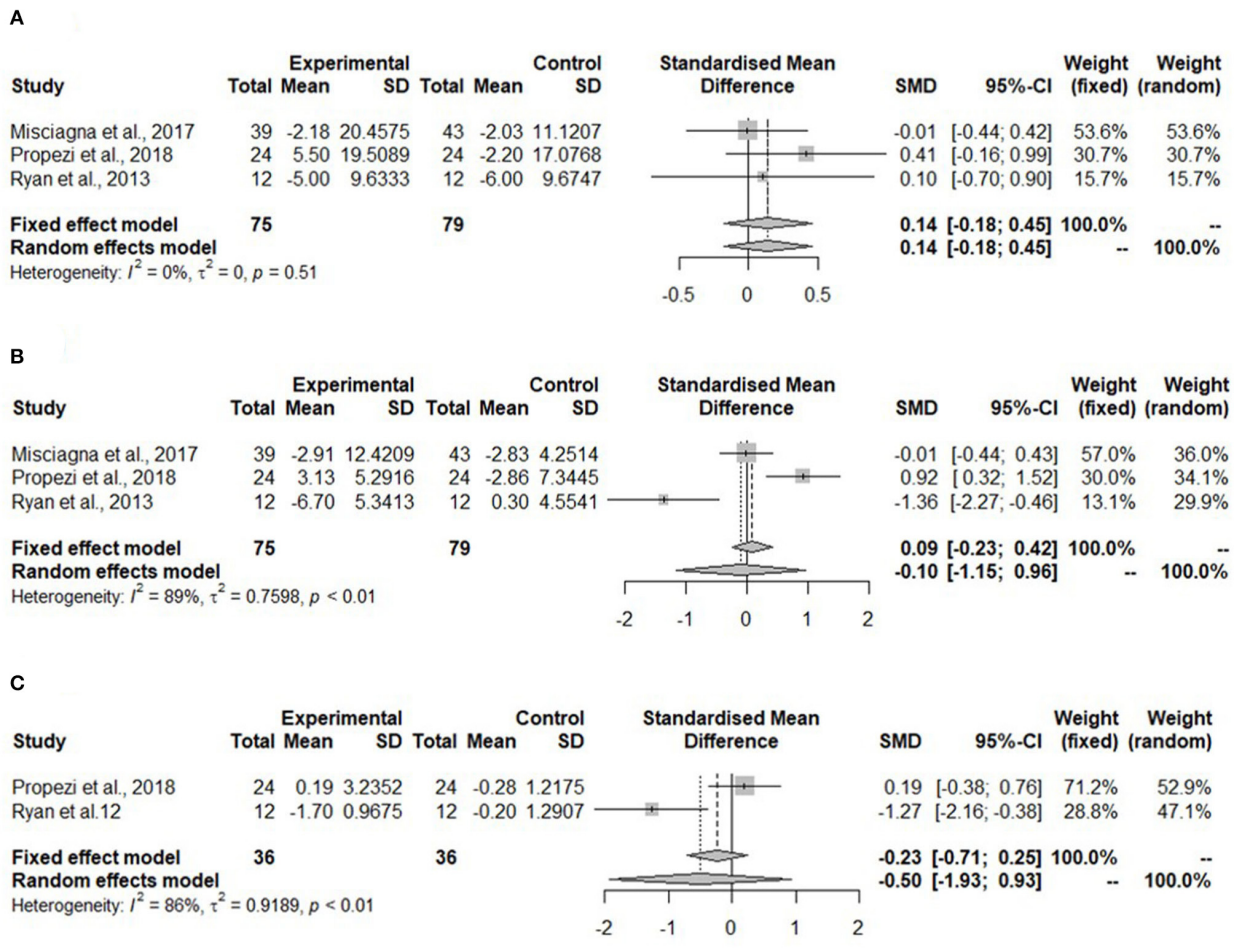
Forest plots of **(A)** fasting glucose in isocaloric diet; **(B)** fasting insulin in isocaloric diet; **(C)** homeostatic model assessment for insulin resistance (HOMA-IR) in isocaloric diet; SD, standard deviation; SMD, standardized mean difference.

Meta-analyses for glucose parameters could be only performed for the Mediterranean diet due to lack of studies on the hypocaloric diet. The meta-analysis in Figure 4A shows non-significant changes in fasting glucose concentration (SMD: 0.14, 95% CI: −0.18, 0.45) after the Mediterranean diet compared to the low-fat diet. Heterogeneity was not detected (*I*^2^ = 0%, τ^2^ = 0, *p* = 0.51).

Meta-analysis for fasting insulin in the Mediterranean diet showed no change (SMD: −0.10, 95% CI: −0.15, 0.96) (Figure 4B). Heterogeneity was significant and high (*I*^2^ = 89%, τ^2^ = 0.7598, *p* < 0.01).

The homeostatic model assessment for insulin resistance was not significantly reduced in the Mediterranean diet (SMD: −0.50, 95% CI: −1.93, 0.93) (Figure 4C). The heterogeneity was high (*I*^2^ = 86%, τ^2^ = 0.9189, *p* < 0.01).

### Lipid Parameters

In the hypocaloric dietary interventions, Shidfar et al. (41) did not measure any lipid parameters and Jang et al. (39) did not measure HDL-C concentration. Razavi et al. (40) reported a decrease in TG concentration (−31.3 ± 38.8 mg/dL, *p* = 0.006) only in the DASH group. Arefhosseini et al. (38) reported a significant decrease of TG concentration in the control group (−39.8 ± 56.5 mg/dl, *p* = 0.023). Jang et al. (39) detected a significant reduction of LDL-C concentration in low carbohydrate intervention arm (−7.1 ± 16.5 mg/dl, *p* < 0.001) compared to the control group. For isocaloricdietary interventions, Ryan et al. (45) did not measure the concentrations of TC and LDL-C. The tendency of the studies with the Mediterranean diet is similar. Compared to its low-fat control group, TC concentration did not change in the Mediterranean diet groups. Dorosti et al. (42) showed significant differences in the concentrations of LDL-C (−12.9 ± 12.0 mg/dL, *p* < 0.05) and HDL-C (1.8 ± 4.2 mg/dL, *p* < 0.05) in the whole-grain diet compared to the group with usual cereals, but after adjusting, no significant changes were observed. Propezi et al. (44) reported significant reductions in TC (−9.6 ± 31.4 mg/dL, *p* = 0.01) and TG (−21.4 ± 48.2 mg/dL, *p* = 0.008) concentrations in the Mediterranean diet. Misciagna et al. (43) performed no statistical tests on lipid parameters.

**Figure 5:**
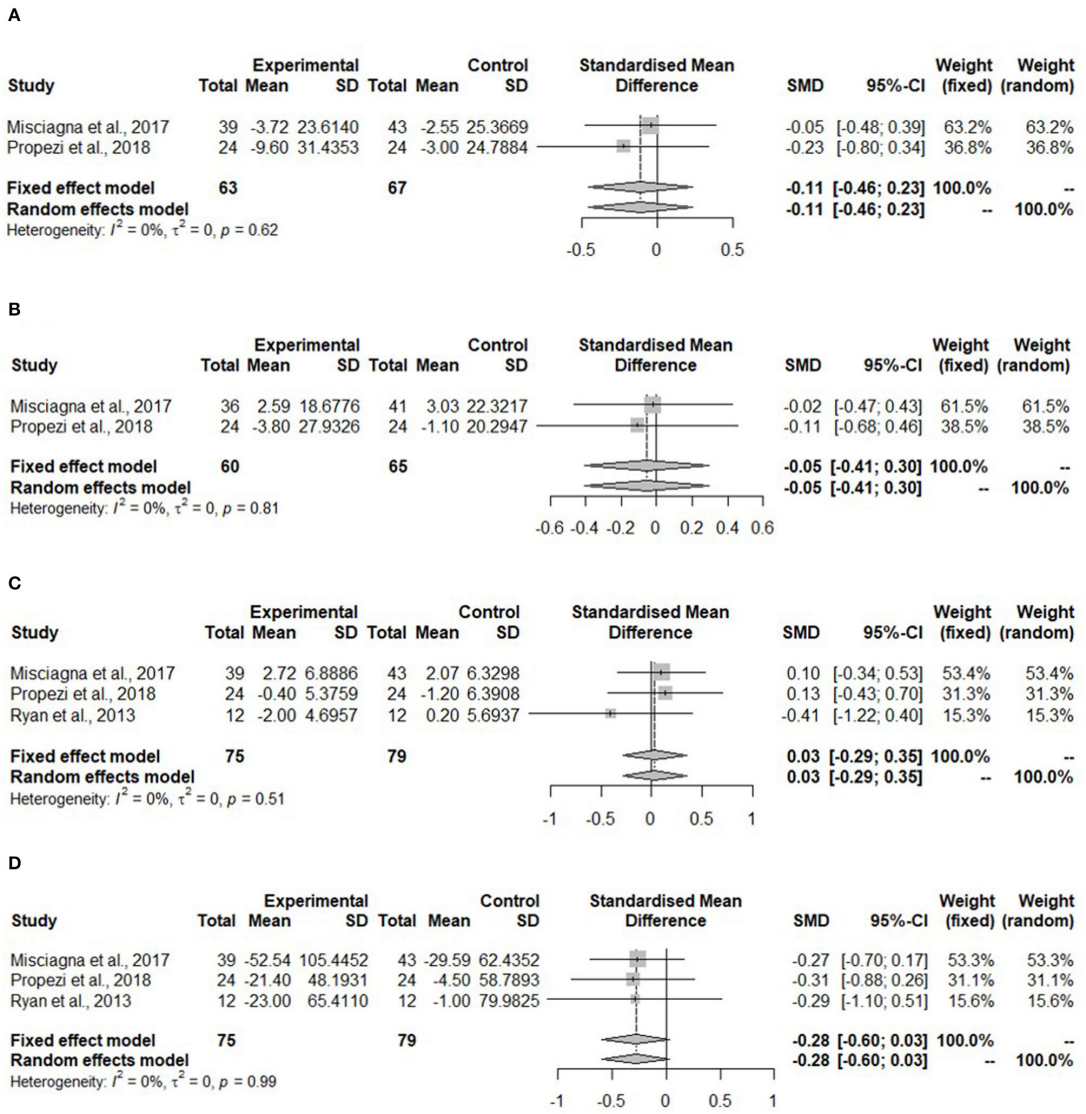
Forest plots of **(A)** total cholesterol (TC) in isocaloric diet; **(B)** low-density lipoprotein cholesterol (LDL-C) in isocaloric diet; **(C)** high-density lipoprotein cholesterol (HDL-C) in isocaloric diet; **(D)** triglycerides (TG) in isocaloric diet; SD, standard deviation; SMD, standardized mean difference.

Meta-analyses for all lipid parameters were only performed in the isocaloric group (Mediterranean group) due to lack of studies in the hypocaloric group (Figure 5). The TC was not significantly reduced in the Mediterranean diet compared to the control group (SMD: −0.11, 95% CI: −0.46, 0.23) and no heterogeneity was detected (*I*^2^ = 0%, τ^2^ = 0, *p* = 0.62) (Figure 5A).

Figure 5B shows no changes in the LDL-C concentration in the Mediterranean diet (SMD:−0.05, 95% CI:−0.41, 0.30). No heterogeneity was detected (*I*^2^ = 0%, τ^2^ = 0, *p* = 0.81). Misciagna et al. (43) had different total subject numbers for this analysis because LDL-C concentration was not measured in all of the subjects.

High-density lipoprotein cholesterol concentration (Figure 5C) showed no changes (SMD: 0.03, 95% CI: −0.29, 0.35). There was no heterogeneity (*I*^2^ = 0%, τ^2^ = 0, *p* = 0.51). The TG concentration did not change in the Mediterranean diet (SMD: −0.28, 95% CI: −0.60, 0.03). There was no heterogeneity (*I*^2^ = 0%, τ^2^ = 0, *p* = 0.99) (Figure 4D).

## Discussion

In this systematic review, the effect of dietary interventions without any exercise or physical activity on NAFLD and related clinical parameters (IHL, ALT, AST, γGT, fasting glucose, fasting insulin, HOMA-IR TC, LDL-C, HDL-C, and TG) were reviewed and analyzed. In total, eight studies fulfilled the inclusion criteria. Dietary interventions were grouped based on the energy intake in the studies resulting in four studies in each group. Further assessment was applied based on the composition of the dietary interventions in order to perform the meta-analysis. This led to the selection of two (hypocaloric dietary interventions) or three (isocaloric dietary interventions) studies for pooled comparisons. Our meta-analysis shows that the Mediterranean diet reduces liver fat in patients with NAFLD, and a hypocaloric dietary approach favoring unsaturated fatty acids reduces liver transaminases as a clinical proxy for NASH; yet no conclusions can be drawn on active or fibrotic NASH.

We were able to replicate the findings from previous systematic reviews investigating the Mediterranean diet on the liver fat (31, 47). The Mediterranean diet without caloric restriction reduced the liver fat compared to the control group in patients with NAFLD. Propezi et al. (44) and Ryan et al. (45) constructed the Mediterranean diet based on the actual reported data on the consumption of a Cretan diet with similar composition of macronutrients from similar sources, whereas the study by Misciagna et al. (43) did not report any basis on the construction of the diet. However, it contained similar macronutrient composition and foods, and they also used a Mediterranean adequacy index (MAI) to follow the adherence to the diet. Based on the information on the diet compositions, the diets in these studies can be considered comparable. In general, the Mediterranean diet is characterized by high proportion of unsaturated fatty acids, including polyunsaturated fatty acids (PUFA) and monounsaturated fatty acids (MUFA) whose main sources are vegetable oils, fish, nuts, and seeds. These can contribute to the beneficial effects on the liver fat (48). For instance, higher consumption of walnuts has been shown to associate with a greater improvement of the liver fat in patients with NAFLD (49). In general, the Mediterranean diet is associated with a 56% decreased risk for NAFLD in a cross-sectional setting (48). Moreover, the Mediterranean diet is also composed of vegetables, fruits, legumes, and whole-grain products that are high in dietary fiber. In fact, the intake of dietary fiber is inversely associated with NAFLD (50). Additionally, the study by Dorosti et al. (42), which was not a part of the meta-analysis, showed a resolution in steatosis, together with a decrease in the liver enzymes after a 12-week whole-grain diet compared to the control diet with usual cereal consumption. This effect may be explained partly due to the fiber content and its effects on the digestion and the gut microbiome, and also due to the properties of phenolic compounds from the whole-grain foods. These phenolic compounds include anthocyanins and flavonoids that have been associated with anti-inflammatory properties and the so called antioxidants (51, 52). However, a supplementation of antioxidants did not give any additional benefits to the Mediterranean diet on the liver fat, but together with green tea, the liver fat improved more significantly than with the diet alone (53). Furthermore, the gut microbiome might mediate some of the metabolic effects of the Mediterranean diet which could contribute to the regression of NAFLD (49, 50, 54). The change in IHL after an 18-month Mediterranean diet was independently associated with the gut microbial compositional changes, depicted in β-diversity and taxa level (49). In general, the diet has distinct impacts on the gut microbiome, and certain gut microbial signatures has been related to NAFLD (55–58). Therefore, investigating this triangle could give more insights on the mechanisms behind the effects of dietary interventions on NAFLD.

Interestingly, the study by Jang et al. (39), one of the hypocaloric dietary interventions, detected a significant decrease in IHL in a low-carbohydrate diet compared to a low-fat diet. However, their low-carbohydrate diet had 50–60% of energy intake as carbohydrates being still in a normal range of carbohydrate intake, and 20–25% of fat which was higher than in the low-fat diet (15–20%). A SR reported that replacing total fats with total carbohydrates does not reduce the liver fat; however, types of carbohydrates were not able to be assessed due to lack of reporting. In turn, replacing saturated fats with unsaturated fats is beneficial to the liver fat (32). Proportion of nutrients toward higher unsaturated fat ratio could contribute to steatosis regression. All individual hypocaloric dietary interventions reported a decrease of IHL together with a significant weight loss but due to the different methods for measuring IHL, an overall effect *via* meta-analysis could not be assessed. Altogether, the dietary fiber and unsaturated fatty acids from foods in the Mediterranean diet can be considered one of the dietary components contributing to the beneficial effects of the Mediterranean diet on the liver fat.

The pooled effect of two hypocaloric diets containing foods high in unsaturated fatty acids show reduction on liver transaminases, such as ALT and AST, compared to the control group (40, 41). We were not able to do meta-analysis on the liver fat with these studies due to the different assessment techniques, but all hypocaloric studies reported a significant decrease in IHL (38–41). Our results of the liver transaminases are in accordance with the previous studies, where a hypocaloric diet improved the liver function through weight loss supporting the beneficial effect of hypocaloric diet *via* weight loss on the liver-related parameters (59, 60). A dose-response relationship between weight loss and improvements in the liver-related outcomes in patients with NAFLD has been reported previously; a hypocaloric diet targeting to 5–7% weight loss reduced steatosis, while over 10% weight loss significantly improved NASH and fibrosis (25, 61). Furthermore, the meta-analysis of the Mediterranean diet showed a tendency toward increased ALT levels. Additionally, the levels of γGT did not show any significant change after the Mediterranean dietary interventions compared to the control diet. Similar results have been reported also by other SR before (40). It is known that γGT is dependent on age, gender, and alcohol consumption (62). However, in all the three studies, high alcohol consumption was an exclusion criterion and it was not changed during the interventions. A non-significant change could be explained by several other factors, such as medication, smoking, and several other diseases, which are all associated with increased γGT (63–65). None of the Mediterranean dietary intervention studies reported significant change in weight between intervention or control group, but studies by Propezi et al. (44) and Misciagna et al. (43) showed significant weight loss within the groups. Regarding the macronutrient composition of dietary interventions, the effect of macronutrient composition on the liver enzymes is still controversial (32). The study by Arefhosseini et al. (38) aimed to address this with two hypocaloric diets with different macronutrient compositions, where both diets were as effective on hepatic outcomes (the liver enzymes and echogenicity) indicating weight loss-induced effects, despite macronutrient composition. In turn, Jang et al. (39) reported greater and significant improvement of ALT in the low-carbohydrate group compared to the low-fat group. Limited number of studies limits our conclusions with respect to the liver enzymes. Overall, hypocaloric dietary interventions in this systematic review showed a significant improvement in the liver enzymes, but our meta-analysis supports dietary interventions with foods high in unsaturated fatty acids.

Our systematic review does not show significant changes in the concentrations of fasting glucose or insulin or HOMA-IR in studies with the Mediterranean diet. Similar results have been reported in other studies, where the Mediterranean diet does not show significant improvements in fasting glucose or insulin concentrations over the control diet, but HOMA-IR, as a surrogate marker for the insulin resistance, showed a significant improvement in another SRs in patients with NAFLD (31, 47). This could be due to the limited number of studies in our SR. However, an improvement of HOMA-IR could indicate a decline in the insulin resistance. Insulin resistance is thought to be the major driver for NAFLD, but it mainly concerns patients without a genetic predisposition, the genetic variant of the gene that encodes patatin-like phospholipase domain-containing protein 3 (PNPLA3) (66, 67). Unfortunately, we were not able to investigate the influence of the genetic variants in our meta-analysis due to lack of data and reported endpoints in the studies. However, individual assessment of the isocaloric studies indicates a significant improvement of HOMA-IR only in one study with the Mediterranean diet (45) and no effect in two studies with either the Mediterranean or a whole-grain diet (42, 44). No significant changes in glucose or insulin concentrations compared to the control diet were reported in either of the isocaloric studies (42–45). None of the hypocaloric dietary interventions showed significant changes in insulin and glucose concentrations (39, 40) or HOMA-IR (40). Ultimately, dietary intervention studies, both hypo and isocaloric, investigating parameters related to glucose metabolism in patients with NAFLD are controversial. More studies are needed in order to assess different dietary approaches to glucose metabolism in patients with NAFLD.

Our meta-analysis shows no significant reduction but a strong trend toward improvement in TG concentrations in isocaloric studies. This trend is in line with other SRs investigating the effect of the Mediterranean diet on cardiovascular risk factors in NAFLD (6, 68). Elevated TGs and their increased ratio to HDL-C are associated with the increased risk of CVD and its outcomes (69, 70), as well as LDL-C concentration is strongly linked to coronary heart disease (71). Moreover, the Mediterranean diet has been linked to reduced risk for cardiometabolic diseases which are thought to be explained by the beneficial effects of vegetable oils, nuts, and seeds which are low in saturated fatty acids and high in unsaturated fatty acids and fiber. It may be also partly due to the effects of phytosterols and phytostanols affecting the intestinal cholesterol absorption (72–74). However, in our pooled analysis with the Mediterranean diet, TC or lipoprotein cholesterol concentrations (HDL-C, LDL-C) did not show significant changes. This is also in line with other SRs that do not report changes in lipoprotein cholesterol concentrations in the Mediterranean diet in patients with NAFLD (47). Moreover, individual assessment of all studies in our review showed that isocaloric dietary interventions can improve plasma lipid profile in patients with NAFLD. The hypocaloric DASH diet with increased intake of foods high in unsaturated fatty acids reported a decrease in concentrations of triglycerides, TC, and LDL-C (40), but unfortunately, pooled effect on lipids could not be investigated with any of the hypocaloric interventions. A clinically significant decrease in TG concentration in the intervention group was shown by Razavi et al. (40). Arefhosseini et al. (38) showed that only a hypocaloric diet with decreased intake of both carbohydrates and dietary fat decreased the concentration of TGs significantly compared to the other diet. Also, Jang et al. (39) reported an increase with a low-carbohydrate diet. Cross-sectional data show that the intake of cholesterol and consumption of foods high in saturated fatty acids have been associated with the risk for NAFLD (50). In general, limiting the intake of unsaturated fatty acids is associated with the lower risk of cardiovascular events (75). As CVD is the main cause of mortality among patients with NAFLD, the quality of dietary fats and other sources of fat, such as meat and dairy, require special attention (76). Additionally, the Western type of dietary pattern high in processed foods, refined grains, and saturated fatty acids from high fat dairy and meat increase the risk for NAFLD by 23% (48).

Currently, dietary modification, exercise, or the combination of both, targeting weight loss is the key therapy for NAFLD (21–24). Our results showed that the Mediterranean diet without weight loss can also improve the liver parameters. Both dietary interventions in this SR are in accordance with EASL, ESPEN, AGA, and AASLD guidelines, where a hypocaloric or the Mediterranean diet is suggested as a dietary treatment for NAFLD. However, current guidelines do not address the optimal dietary strategy specifically for active, fibrotic, and cirrhotic NASH, although it contributes to the overall burden of the disease, not only on the t point of view of patients but also economically on the public health (77–80). Due to this burden, different stages, especially active and fibrotic NASH, cannot be disregarded when designing future studies. Furthermore, based on the knowledge of the distinct metabolic effects of genetic variants on the pathophysiology on NAFLD, the genetic predispositions should have more attention in lifestyle interventions. We suggest that more RCTs with different comparable dietary intervention studies including well-characterized NAFLD patients and valid endpoint measures, including histology and/or multiparametric imaging are warranted in order to identify and address the optimal dietary approaches in the future guidelines. This would help clinicians to develop care pathways and target their resources efficiently in the different stages of NAFLD. However, compliance and successful treatment require that the patient is motivated and engaged to the lifestyle modifications (81, 82). Therefore, the optimal treatment of NAFLD should be patient-centered and multidisciplinary (83). In order to achieve these goals, we need more awareness from the policy, care pathway, and guideline creators (83, 84). Additionally, NAFLD should be incorporated in the national health prevention and care strategies due to its large public health burden.

One of the main strengths of this SR is the study inclusion criteria, which resulted in a well-characterized study population focusing on NAFLD. Also, studies were limited to dietary interventions without any kind of exercise or physical activity intervention. To the best of our knowledge, this has not been done before in SRs investigating dietary interventions in patients with NAFLD. Moreover, the quality assessment was conducted by a tool which is specifically designed for dietary intervention studies in a three-category grading system. This allows more detailed and valid assessment of the quality of the studies. However, this paper has also some limitations. First, due to the small number of studies, meta-analyses were limited. In addition, most of the studies were performed in Iran which could cause some bias. Therefore, the reliability of the results should be interpreted with some caution. With a higher number of studies, it would be probably possible to show more robust effects of different dietary interventions in several parameters. Consequently, a publication-bias detection analysis could not be performed due to the lack of power with a small number of studies. Another limitation is the missing genetic predispositions. Genetic variants should be assessed in dietary intervention studies since the presence of those variants affect lipid and glucose metabolism. Moreover, most of the studies do not report the severity and the activity of the disease which potentially affects the endpoints. Thus, by reporting this and its influence on the results could improve the analysis.

In conclusion, the Mediterranean and hypocaloric dietary interventions favoring unsaturated fatty acids result in improvements in IHL independent of weight loss, and transaminases are reduced in the hypocaloric diets with weight loss in patients with NAFLD. Since many dietary intervention studies are combined with exercise interventions or physical activity, the diet effect alone is not investigated enough. In addition, to the best of our knowledge, studies addressing the genetic background in diet interventions are missing. Furthermore, more advanced and clinically relevant stages of NAFLD, that is active and fibrotic NASH, with multiparametric imaging and liver histology as outcome measures, are not sufficiently considered. Therefore, the optimal dietary invention in NAFLD remains to be defined.

## Data Availability Statement

Publicly available datasets were analyzed in this study. This data can be found at: The data is retrieved from the listed articles in the manuscript.

## Author Contributions

SC and VH contributed equally to the organization as well as in writing, reviewing, and editing of the manuscript. Data extraction and quality assessment were also performed by SC and VH with the assistance of US. SC performed the statistical analyses for the meta-analysis. MN, AH, and US reviewed, edited, and supervised the manuscript. All authors contributed to the article and approved the submitted version.

## Conflict of Interest

The authors declare that the research was conducted in the absence of any commercial or financial relationships that could be construed as a potential conflict of interest.
